# Cholesteryl Ester Species but Not Serum Proprotein Convertase Subtilisin/Kexin Type 9 Levels Decline in Male Patients with Active Inflammatory Bowel Disease

**DOI:** 10.3390/pathophysiology32020013

**Published:** 2025-03-25

**Authors:** Angelika Hettenbach, Tanja Elger, Muriel Huss, Gerhard Liebisch, Marcus Höring, Johanna Loibl, Arne Kandulski, Martina Müller, Hauke Christian Tews, Christa Buechler

**Affiliations:** 1 Department of Internal Medicine I, Gastroenterology, Hepatology, Endocrinology, Rheumatology, Immunology, and Infectious Diseases, University Hospital Regensburg, 93053 Regensburg, Germanytanja.elger@klinik.uni-regensburg.de (T.E.); muriel.huss@klinik.uni-regensburg.de (M.H.); johanna.loibl@klinik.uni-regensburg.de (J.L.); arne.kandulski@klinik.uni-regensburg.de (A.K.); martina.mueller-schilling@klinik.uni-regensburg.de (M.M.); hauke.tews@klinik.uni-regensburg.de (H.C.T.); 2 Institute of Clinical Chemistry and Laboratory Medicine, University Hospital Regensburg, 93053 Regensburg, Germany; gerhard.liebisch@klinik.uni-regensburg.de (G.L.); marcus.hoering@klinik.uni-regensburg.de (M.H.)

**Keywords:** ulcerative colitis, fecal calprotectin, diarrhea, cholesterol

## Abstract

Background/Objectives: Proprotein convertase subtilisin/kexin type 9 (PCSK9) regulates serum cholesterol levels and inflammation, both of which are dysregulated in inflammatory bowel disease (IBD). Free cholesterol (FC) and the various types of cholesteryl ester (CE) have different functions in the body. However, it is not yet known whether these lipids undergo parallel changes in male and female patients with active IBD, nor whether PCSK9 correlates with these lipids and disease severity in either sex. The present study measured the serum levels of PCSK9, FC, and 15 CE species in IBD patients, focusing on the associations of these molecules with sex, each other, and with disease severity. Methods: The serum PCSK9 levels of 80 IBD patients (42 males and 38 females) and 24 controls (12 males and 12 females) were measured by enzyme-linked immunosorbent assay. In addition, FC and 15 CE species levels of 53 randomly selected IBD patients and 16 controls were determined by direct flow injection analysis (FIA) using a high-resolution hybrid quadrupole-orbitrap mass spectrometer (FIA-FTMS). Results: Serum PCSK9 levels in controls and IBD patients were comparable and did not correlate with disease severity in IBD patients. There was no discernible difference in serum PCSK9, FC, and CE levels between patients with Crohn’s disease (CD) and those with ulcerative colitis (UC). FC and almost all CE species decreased in male patients with active IBD but were not related to disease severity in the female patients. The decrease in different CE species in male IBD patients with diarrhea compared to those with normal stool consistency appears to be related to IBD severity. Bile acids regulate serum cholesterol levels, and FC and CE levels were positively correlated with fecal levels of secondary bile acids in the patients with UC but not CD. This association also existed in male UC patients and could not be evaluated in women due to the small sample size. Conclusions: In active IBD, a reduction in FC and almost all CE species was observed only in males, while serum PCSK9 levels remained within normal ranges in both sexes. It can be hypothesized that blocking PCSK9 may further reduce serum cholesterol levels, which may have adverse effects in male patients with active IBD.

## 1. Introduction

Systemic inflammation is associated with low serum cholesterol levels, which also declines in patients with active inflammatory bowel disease (IBD) [1,2]. Crohn’s disease (CD) and ulcerative colitis (UC) are the most common IBD entities [3,4], and the etiologies of both remain unclear [5]. The same is true for the mechanisms that cause cholesterol to fall in systemic inflammation and active IBD [2,6,7].

Cholesterol homeostasis is tightly regulated and the major transcription factor for cholesterol synthesis, sterol regulatory element-binding protein 2 (SREBP2), is activated upon cholesterol depletion [8]. It has been shown that the levels and activity of SREBP2 are reduced in the inflamed colon of patients with IBD and that the response of these cells to inflammatory stimuli is enhanced [9]. However, it is unclear to what extent intestinal cholesterol synthesis contributes to hypocholesterolemia in active IBD.

Dietary cholesterol is taken up as free cholesterol (FC) and is esterified by intestinal acyl-coenzyme a cholesterol acyltransferase 2 (ACAT) 2. ACAT2 is also expressed in the liver and produces predominantly saturated and monounsaturated cholesteryl ester (CE) species [10,11,12]. Lecithin cholesterol acyl transferase (LCAT) is responsible for the synthesis of polyunsaturated CE species in plasma. It is generally accepted that n-3 polyunsaturated fatty acids are healthier than saturated fats and that reducing CE production by ACAT may protect against atherosclerosis [13]. Accordingly, there is a shift from polyunsaturated to saturated CEs in diseases such as coronary artery disease [14], chronic hepatitis C virus infection, and liver cirrhosis [15]. Patients with IBD have a higher risk for atherosclerosis [16]. Whether there is a shift from unsaturated to saturated CE species in patients with IBD has not been analyzed to our knowledge. Preliminary data also suggest that cholesteryl linoleate and cholesteryl arachidonate may inhibit bacterial growth, further pointing to specific functions of individual CE species [17].

Cholesterol levels differ between the sexes, largely reflecting the effect of sex hormones [18], which also have a role in IBD pathogenesis [19]. Total cholesterol levels in men and women with IBD were similar, with women having lower low-density lipoprotein (LDL) and higher high-density lipoprotein levels, as seen in the normal population [20,21]. To the best of our knowledge, no published studies have compared serum cholesterol levels between silent and active disease in either sex.

Proprotein convertase subtilisin/kexin type 9 (PCSK9) levels, which correlate positively with LDL levels, are higher in females than males in the general population [22]. LDL receptor degradation is induced by PCSK9 binding, and the inhibition of PCSK9 increases receptor recycling and the liver uptake of systemic cholesterol, thereby lowering serum LDL cholesterol [23]. In addition to its role in cholesterol metabolism, PCSK9 has been shown to regulate inflammatory pathways. Increased PCSK9 levels in sepsis are thought to contribute to the severity of the disease through mechanisms that have yet to be fully elucidated [23]. The serum levels of PCSK9 are also elevated in patients with active UC. In these patients, serum PCSK9 was positively correlated with fecal calprotectin, C-reactive protein, and the Mayo endoscopic score [24]. This study also reported a positive correlation between PCSK9 and both total and LDL cholesterol. However, almost 60% of these UC patients had hypercholesterolemia, which is associated with higher levels of PCSK9 [24,25].

An adenovirus-mediated experimental approach to block colonic PCSK9 improved inflammation and colitis severity in rats, suggesting a functional link between high PCSK9 protein levels in the colon of these animals and disease severity. Specifically, this study described higher PCSK9 protein levels in the colon of rats with IBD compared to control animals [26]. To date, no studies have been conducted to measure PCSK9 in the intestinal tissue of control subjects and patients with IBD.

Whether systemically administered antibodies that block PCSK9 protect against severe inflammation in IBD has not been studied. PCSK9 antibody therapy was effective in SARS-CoV-2 infection but did not protect against infection and inflammation in sepsis [27,28,29]. Mendelian randomization analysis failed to detect an association between PCSK9 inhibition and IBD risk and even described an increased risk of IBD with this intervention [30,31,32].

The majority of studies have analyzed both LDL cholesterol and total systemic cholesterol levels in IBD [7]. It is now clear that the different types of CE and FC have different functions [13,33,34]. In addition, the sex differences in cholesterol levels [18] have been largely ignored. To better understand the association of FC and CE species levels as well as PCSK9 in IBD severity while also considering that FC and CE species vary in abundance and have different roles [34,35,36,37], this study measured serum PCSK9, FC, and 15 CE species in male and female patients with IBD.

## 2. Materials and Methods

### 2.1. Study Cohorts

From 12 June 2021 to 23 February 2023, patients admitted to a German university hospital with a confirmed diagnosis of IBD were invited to participate in a study. IBD was diagnosed based on accepted endoscopic, histological, and clinical criteria [38,39]. Classification into UC and CD was based on imaging, endoscopic, serological, and histopathological criteria. Individual disease phenotypes can be clearly distinguished from each other, and only in some cases is the distinction between UC and CD unclear, so that classification can only be made later in the course of the disease or not at all [40,41]. Only patients with a known phenotype of the disease were included in the study. Females who were pregnant and patients who had coagulopathy or were unable to give informed consent were excluded from the study. Patients with primary sclerosing cholangitis were also excluded. Control subjects included students, hospital staff, and patients’ spouses. All controls were found to be healthy and had a normal weight.

The study was approved by the Ethics Committee (Protocol No. 19-1309-101, Approval date: 20 February 2019) and all participants gave written informed consent to the study. The latest versions of good clinical practice guidelines and the updated Declaration of Helsinki were followed in the trial.

Blood samples were taken at the same time for analysis of routine laboratory parameters, for analysis of lipids and PCSK9.

### 2.2. ELISA

The DuoSet ELISA for human PCSK9 (order number DY3888) was purchased from Bio-Techne GmbH (Wiesbaden, Nordenstadt, Germany) and utilized in accordance with the company’s guidelines. The serum was diluted to a ratio of 1:100. Samples were measured twice, and the mean value was used for calculations. Spot urine was collected and utilized without dilution for the ELISA assay. Urinary creatinine levels were determined using the Creatinine Parameter Assay Kit (order number KGE005; Bio-Techne GmbH, Minneapolis, MN, USA) in 1:20 diluted urine.

### 2.3. Measurements of Serum Free Cholesterol and Cholesteryl Ester Levels and Fecal Bile Acids

Lipids were isolated from 10 µL of serum as described [42]. The non-natural internal standards were added to the serum prior to lipid extraction. Cholesteryl ester (CE) levels were determined by direct flow injection analysis (FIA) using a high-resolution hybrid quadrupole-Orbitrap mass spectrometer (FIA-FTMS) as described [43]. CE was recorded at *m/z* 500–1000 as [M+NH_4_]^+^ in positive ion mode with a target resolution of 140,000 (at 200 *m/z*). CE species were corrected for their specific responses [44]. FC levels were determined by multiplexed acquisition of the [M+NH_4_]^+^ of FC and the deuterated internal standard (FC[D7]) [44]. The levels of bile acids in feces in patients with IBD and control subjects and the methodology used have been described previously [45,46].

### 2.4. Immunoblot

The immunoblot analysis of urine was conducted in accordance with the established protocol [47]. The PCSK9 antibody (order number 55728), procured from Cell Signaling (Danvers, MA, USA), was diluted at a ratio of 1:500 fold for the purpose of PCSK9 detection.

### 2.5. Examination of Laboratory Parameters

Laboratory parameters were analyzed at the time the patient gave blood for analysis of PCSK9 and cholesterol levels. C-reactive protein (CRP) levels were determined by a particle-enhanced immunoturbidimetric assay. Aspartate aminotransferase (AST) catalyzes the transfer of an amino group between L-aspartate and 2-oxoglutarate, resulting in the formation of oxaloacetate and L-glutamate. Oxaloacetate then interacts with malate dehydrogenase and the reduced form of nicotinamide adenine dinucleotide (NADH) to produce the oxidized form of this coenzyme (NAD+) and L-malate. The rate of oxidation of NADH is determined by measuring the quenching rate and is proportional to the catalytic activity of AST. Alanine aminotransferase (ALT) facilitates the transfer of an amino group from L-alanine to 2-oxoglutarate to form pyruvate and L-glutamate. Pyruvate then reacts with NADH in the presence of lactate dehydrogenase to form L-lactate and NAD+. The rate of oxidation of NADH is measured as described above. Total bilirubin is measured by coupling it with 3,5-dichlorophenyldiazonium in an acidic medium. The intensity of the resulting red azo dye is directly related to the total bilirubin concentration and can be quantified by photometric methods. Alkaline phosphatase (AP) cleaves the substrate p-nitrophenyl phosphate to release phosphate and p-nitrophenol, which is measured photometrically. Gamma-glutamyltransferase (GGT) transfers the gamma-glutamyl group of L-gamma-glutamyl-3-carboxy-4-nitroanilide to glycylglycin to form 5-amino-2-nitrobenzoate, which is quantified photometrically. All of these assays described above were supplied by Roche (Penzberg, Germany) and performed on the Cobas Pro C analyzer.

Fecal calprotectin levels were determined using the Quanta Flash Calprotectin assay from Inova Diagnostics (order number 701350; San Diego, CA, USA). This chemiluminescence sandwich immunoassay is performed on the Bio-Flash instrument.

### 2.6. Statistical Analysis

The data are presented as a box plot, also known as a box and whisker plot. The line dividing the box in two is the median. This indicates that 50% of the data are below the median and the remaining 50% is above it. The lower boundary of the box indicates the lower quartile, which is the value below which the first 25% of the data fall. Conversely, the upper boundary of the box indicates the upper quartile, indicating that 25% of the data are above this upper quartile value. The endpoints of the horizontal lines correspond to the minimum and maximum values of the data set. The individual points/asterisks shown in the graph represent the outliers.

PCSK9 levels in serum and urine, body mass index, fecal calprotectin, CRP, CE 14:1, 15:1, 20:5, 22:5, and 22:6 were not normally distributed, as assessed by the Kolmogorov–Smirnov and Shapiro–Wilk tests. The non-parametric test can be applied to all types of data [48] and so non-parametric tests were applied. Statistical differences were evaluated using the Wilcoxon test, the Mann–Whitney U test, the Kruskal–Wallis test, and the chi-square test. When using the Kruskal–Wallis test, Bonferroni correction is automatically applied by the software for significant *p*-values. The relationship between the two measurements was assessed using Spearman’s correlation (IBM SPSS Statistics 26.0; IBM Corp., Armonk, NY, USA, released 2019). A *p*-value of <0.05 was considered significant.

## 3. Results

### 3.1. Serum PCSK9 Levels of Controls and Patients with IBD

Serum PCSK9 levels were measured in 80 patients with IBD and 24 controls (12 males 54.0 (22.9–78.1) years old; *p* = 0.432 compared to IBD patients) and 12 females (51.3 (23.4–65.6) years old; *p* = 0.220 compared to IBD patients). Control subjects included students, hospital staff, and patients’ spouses. All of the controls were in good health and had normal body weights, and the laboratory values were not recorded. Serum lipids were measured in 53 randomly selected patients and 16 controls (7 males and 9 females, 52.8 (29.3–78.1) years old).

Differences in cholesterol levels between women and men are observed in the general population [21], and a sex-specific analysis was performed. Details of the patients are summarized in Table 1. Men had higher gamma glutamyl transferase and bilirubin levels than women in both cohorts (Table 1). All other laboratory values were comparable between the sexes.

Gender disparities for PCSK9 were non-existent in both the control group (*p* = 0.184) and in the IBD cohort (*p* = 0.494). Serum PCSK9 demonstrated a positive correlation with age in the IBD cohort (r = 0.287, *p* = 0.010), yet this association was not significant in the control group (r = 0.133, *p* = 0.537). In female patients the correlation of PCSK9 with age (r = 0.416, *p* = 0.009) was significant. In males, this correlation was not significant (r = 0.141, *p* = 0.378).

**Figure 1:**
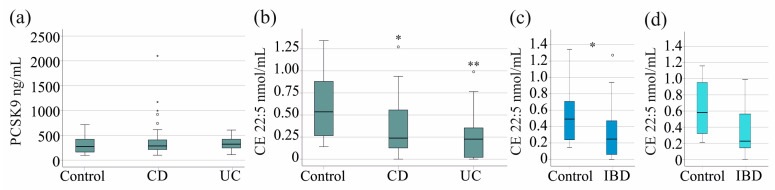
Serum levels of PCSK9 and cholesteryl ester (CE) 22:5 of patients and controls. (**a**) Serum PCSK9 levels of controls, patients with Crohn’s disease (CD), and patients with ulcerative colitis (UC); (**b**) serum levels of CE 22:5 of controls, patients with CD, and patients with UC; (**c**) serum levels of CE 22:5 of male controls and male IBD patients; (**d**) serum levels of CE 22:5 of female controls and female IBD patients. * *p* < 0.05, ** *p* < 0.01 in comparison to controls. All other CE species measured were similar between these cohorts. Statistical test: Kruskal–Wallis test with post hoc Bonferroni test in (**a**,**b**) and Mann–Whitney U test in (**c**,**d**). Small circles and asterisks in the figures are outliers.

A comparison of the serum PCSK9 levels in the control and IBD cohorts revealed no significant differences in the entire cohort (*p* = 0.371) for males (*p* = 0.124) and females (*p* = 0.856). Furthermore, a comparative analysis of the serum PCSK9 levels among the 24 controls, 51 patients with CD, and 29 UC patients revealed no significant differences (*p* = 0.551, Figure 1a). It was not possible to perform sex-specific analyses in patients with CD and UC due to the small sample size.

### 3.2. Correlation of PCSK9 with Free Cholesterol and Cholesteryl Ester Levels

In the present study, a total of 53 patients suffering from IBD and 16 healthy individuals were selected for the purpose of conducting a lipid analysis of their serum. All of our controls had total cholesterol levels below 6.2 mmol/L and did not have hypercholesterolemia [49]. In the patient cohort, seven patients (9%) had hypercholesterolemia.

The total cholesterol and the FC levels of the patients and controls were found to be similar in the entire cohort, between male controls and patients, and between female controls and patients (*p* > 0.05 for all). There was no sex-specific difference for FC and CE species levels in patients with IBD. However, a reduction in the level of the polyunsaturated CE species CE 22:5 was observed in patients with IBD (*p* = 0.007). This reduction was observed in both CD and UC (Figure 1b). The remaining CE species did not differ between the controls and IBD patient group. CD and UC patients had similar levels of these CE species (*p* > 0.05). A sex-specific analysis showed the decline of CE 22:5 in both sexes, which was significant in males (Figure 1c,d).

In IBD, a positive correlation was observed between serum PCSK9 levels and total cholesterol levels (Table S1). Consequently, positive associations of serum PCSK9 with CE 14:0, 14:1, 15:0, 15:1, 16:1, 18:1, 18:3, 20:3, and FC were identified (Table S1). A sex-specific analysis revealed that CE 14:0, 14:1, 15:1, 16:1, 18:3, and FC correlated with PCSK9 in males but not females (Table 2).

### 3.3. Correlation of PCSK9, FC, and CE Species with Measures of Inflammation

Serum PCSK9 did not demonstrate a correlation with fecal calprotectin and C-reactive protein (CRP) in IBD patients in the entire cohort, in both male and female patients (Tables S1 and 2). Furthermore, no significant correlation was found between serum PCSK9 and fecal calprotectin levels in either CD (*p* = 0.611) or UC (*p* = 0.899).

With the exception of CE 14:1 and 16:1, all other CE types exhibited a negative correlation with CRP, while all but CE 22:4 negatively correlated with fecal calprotectin in the entire cohort (Table S1). Furthermore, both fecal calprotectin and CRP levels exhibited a negative correlation with both FC and total cholesterol levels (Table S1).

In male patients with IBD, with the exception of CE 14:1, 15:1, and 16:1, all other CE types exhibited a negative correlation with CRP, and all but CE 22:4 and CE 22:6 negatively correlated with fecal calprotectin (Table 2). In male patients, fecal calprotectin and CRP levels exhibited a negative correlation with FC and total cholesterol levels (Table 2). In females, the negative correlations between CE 18:2, 18:3, FC, and total cholesterol with fecal calprotectin were significant (Table 2).

### 3.4. PCSK9 and Cholesteryl Ester Levels in Relation to Fecal Calprotectin Levels

In our study group, we identified 17 male patients (12 for CE) who had fecal calprotectin levels below 50 µg/g. Ten male patients (six for CE) had levels ranging from 50 to 150 µg/g, while five male patients (four for CE) fell between 150 and 500 µg/g. Eight male patients (six for CE) had fecal calprotectin levels exceeding 500 µg/g. The data of two patients were not recorded.

There were 20 female patients (12 for CE) who had fecal calprotectin levels below 50 µg/g. Nine female patients (eight for CE) had levels ranging from 50 to 150 µg/g, while seven female patients (three for CE) fell between 150 and 500 µg/g. Two female patients (one for CE) had fecal calprotectin levels exceeding 500 µg/g.

**Figure 2:**
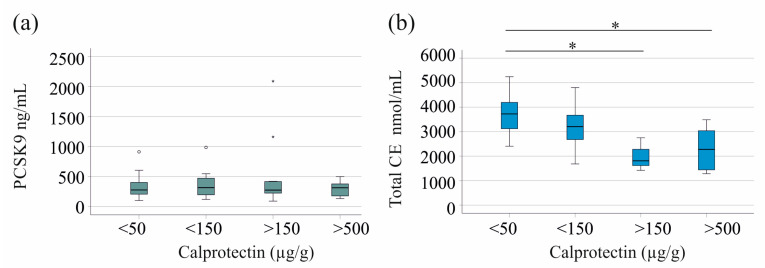
PCSK9 and cholesteryl ester (CE) levels in relation to fecal calprotectin. (**a**) Serum PCSK9 of patients with IBD classified for fecal calprotectin levels; (**b**) total CE serum levels of male patients with IBD classified for fecal calprotectin levels. <50: calprotectin levels below 50 µg/g, <150: calprotectin levels from 50 to 150 µg/g, >150: calprotectin levels between 150 and 500 µg/g, >500: calprotectin levels exceeding 500 µg/g. * *p* < 0.05 Statistical test: Kruskal–Wallis test with post hoc Bonferroni test. Small circles and asterisks in the figures are outliers.

Notably, the PCSK9 levels remained consistent across these categories in the whole cohort (see Figure 2a) and when analyzed for males (*p* = 0.765) and females (*p* = 0.851). Interestingly, male patients with fecal calprotectin levels between 150 and 500 µg/g, as well as those over 500 µg/g, demonstrated lower total CE (Figure 2b) and FC (*p* = 0.016 and *p* = 0.037, respectively) levels compared to patients with levels below 50 µg/g. There was no significant decrease in FC and total CE levels in female patients with severe IBD.

**Figure 3:**
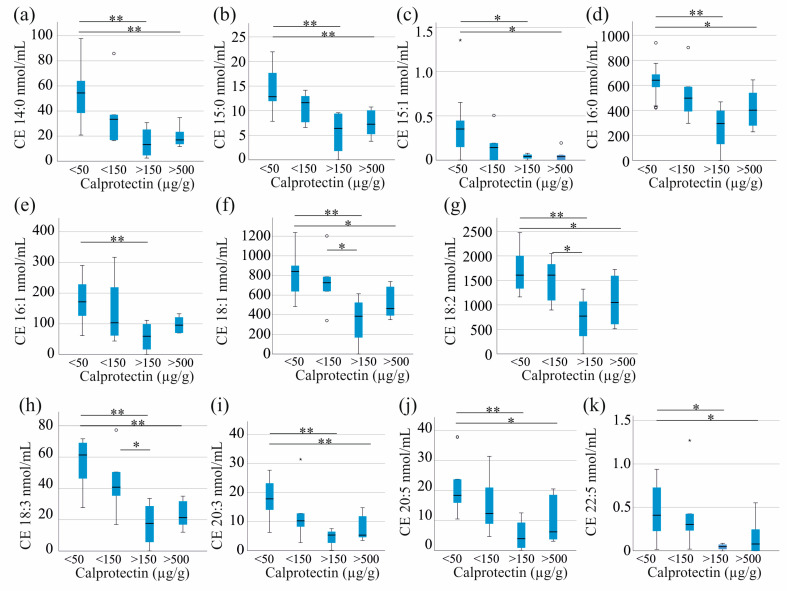
Cholesteryl ester (CE) levels in relation to fecal calprotectin of male patients. (**a**) CE 14:0; (**b**) CE 15:0; (**c**) CE 15:1; (**d**) CE 16:0; (**e**) CE 16:1, (**f**) CE 18:1; (**g**) CE 18:2; (**h**) CE 18:3; (**i**) CE 20:3; (**j**) CE20:5 and (**k**) CE 22:5 of male patients with IBD classified for fecal calprotectin levels <50: calprotectin levels below 50 µg/g, <150: calprotectin levels from 50 to 150 µg/g, >150: calprotectin: levels between 150 and 500 µg/g, >500: calprotectin levels exceeding 500 µg/g. * *p* < 0.05, ** *p* < 0.01. Statistical test: Kruskal–Wallis test with Bonferroni correction. Small circles and asterisks in the figures are outliers.

CE 14:1. 20:4, 22:4 and 22:6 did not change with higher fecal calprotectin levels. Except CE 16:1 all other CE types were reduced in male patients with the highest fecal calprotectin levels in comparison to those with the lowest levels (Figure 3). In addition, all of these CE species were diminished in patients with fecal calprotectin levels ranging from 150 to 500 µg/g, in contrast to those with levels below 50 µg/g. Specifically, CE 18:1, 18:2, and 18:3 levels were lower in patients with fecal calprotectin levels between 150 to 500 µg/g and those with levels between 50 and 150 µg/g (Figure 3).

In females, a significant decline of CE species levels with higher calprotectin was not observed.

### 3.5. PCSK9, Free Cholesterol, and Cholesteryl Ester Levels in Relation to Disease Localization

In patients diagnosed with CD, the distribution of the disease was determined as ileocecal in 10 patients, ileocecal with additional gastrointestinal involvement in 38 patients, and colonic in 3 patients. Subsequent analysis revealed no significant differences in serum PCSK9 levels among these groups (*p* = 0.362). A similar outcome was observed in the association between serum CE species and FC levels and disease localization (*p* > 0.05 for all).

In the UC group, disease localization included pancolitis (18 patients), left-sided colitis (5 patients), proctosigmoiditis (4 patients), and proctitis (1 patient), with one patient’s localization being undocumented. Once more, no significant differences were observed in serum PCSK9, FC, or CE species levels across these groups (*p* > 0.05 for all). Due to the small number of patients in each group, an analysis according to sex was not carried out.

### 3.6. Relation of PCSK9 and Cholesterol with Bristol Stool Chart and Gastrointestinal Symptom Rating Scale

Stool consistency of the patients was evaluated using the Bristol Stool Chart, a system that categorizes stool consistency into seven types. Types 1 and 2 of the chart indicate constipation (affecting one male patients in the whole cohort and one in the cohort for lipid analysis), types 3 and 4 denote normal stool consistency (12/9 patients), types 5 and 6 indicate diarrhea (20/15 patients), and type 7 indicates watery stool (5/3 patients). Constipation affected three female patients in the whole cohort and three in the cohort for lipid analysis, normal stool consistency (7/7 patients), diarrhea (17/10 patients), and watery stool (4/2 patients). The data of 11 patients were not recorded.

The analysis revealed statistically significant association between total serum cholesterol levels as well as FC levels and stool consistency in males. CE 16:0, CE 18:1, CE 18:2, CE 18:3, CE 20:3, CE 20:4, CE 20:5, CE 22:4, and CE 22:6 negatively correlated with the Bristol Stool Score (Table S2).

Male patients suffering from diarrhea had lower serum levels of FC (*p* = 0.026), CE (*p* = 0.009), and total cholesterol (*p* = 0.014) in comparison to those exhibiting normal stool consistency. CE 18:1 (*p* = 0.023), CE 18:2 (*p* = 0.001), and CE 22:6 (*p* = 0.007) were low in male patients with diarrhea in comparison to those exhibiting normal stool consistency. Conversely, fecal calprotectin (*p* = 0.020) and CRP (*p* = 0.009) levels exhibited an increase in cases of less stool consistency.

FC and CE levels did not correlate with the Bristol stool score in females (Table S2) Female patients suffering from diarrhea had lower serum levels of CE 14:0 (*p* = 0.016), CE 14:1 (*p* = 0.029), and CE 15:0 (*p* = 0.029) in comparison to those exhibiting normal stool consistency. Total FC levels (*p* = 0.442), CE levels (*p* = 0.290), and total cholesterol (*p* = 0.399) were all normal. Fecal calprotectin (*p* = 0.084) and CRP levels (*p* = 0.012) exhibited an increase in cases of less stool consistency.

The Gastrointestinal Symptom Rating Scale (GSRS) is a tool designed for the evaluation of gastrointestinal symptoms. There was 1 male patient who had no complaints (1 patient for CE analyses), 24 (16) minor, 12 (9) moderate, and 2 (1) strong complaints. The data of three male patients were not recorded. There was 1 female patient who had no complaints (1 patient for CE analyses), 21 (16) minor, 14 (6) moderate, and 2 (1) strong complaints. In our entire cohort and in females, no significant associations were identified between PCSK9, FC, CE species levels, CRP, fecal calprotectin, and the GSRS (Table S2). In male patients, all but CE 16:1, 20:4, and CE 22:6 negatively correlated with the GSRS score (Table S2). CRP (r = 0.455, *p* = 0.005) and fecal calprotectin (r = 0.438, *p* = 0.005) positively correlated with the GSRS in males.

### 3.7. Effects of Medication on PCSK9 and Cholesteryl Ester Levels

The present therapeutic intervention may exert an effect on CE levels and composition [50], which was also analyzed. A total of 26 patients (13 males and 13 females (17 for CE analysis with 9 males and 8 females)) received anti-TNF antibodies, while corticosteroids were administered to 22 patients (9 males and 13 females (13 for CE analysis with 6 males 7 females)), and anti-interleukin (IL)-12/23 antibody therapy was provided to 21 (11 males and 10 females) (12 for CE analysis with 7 males and 5 females) patients. These drugs did not demonstrate any associations with PCSK9, FC, total cholesterol, and CE species levels in the entire cohort, both male and female patients with IBD (*p* > 0.05 for all).

### 3.8. Correlation of PCSK9 and CE Species with Laboratory Values of Liver Disease

An investigation was conducted into associations related to liver function. The analysis revealed no significant correlations between serum PCSK9 levels and the liver enzymes alanine aminotransferase (ALT), aspartate aminotransferase (AST), gamma-glutamyl transferase (GGT), alkaline phosphatase, or bilirubin in the entire cohort (Table S3). Additionally, in the entire cohort, no connections were found between total cholesterol levels or CE species levels and the liver laboratory tests (Table S3).

In males, positive correlations between CE 18:2 and AP (r = 0.413, *p* = 0.036) as well as CE 20:3 and ALT (r = 0.432, *p* = 0.031) and GGT (r = 0.473, *p* = 0.015) were observed. CE 40:4 correlated with ALT (r = 0.405, *p* = 0.045) and AP (r = 0.451, *p* = 0.021). In females, CE 15:0 correlated with ALT (r = −0.470, *p* = 0.027) and CE 18:2 (r = −0.491, *p* = 0.020), and CE 22:6 (r = −0.564, *p* = 0.006) correlated with GGT.

### 3.9. Serum Cholesterol and Fecal Bile Acids

Bile acids are important for cholesterol excretion, and fecal bile acid levels of this cohort have recently been described [46,51,52]. Dysregulated fecal bile acids were found in UC but not CD [46]. Therefore, the correlations of serum FC and CE levels with primary and secondary fecal bile acids were analyzed separately for UC (16 patients) and CD (33 patients) (Table 3). Total fecal bile acid levels and secondary bile acid levels were positively correlated with FC, CE, and total cholesterol in UC but not in CD patients (Table 3). In the 12 male UC patients, the correlation of secondary bile acids with total CE (r = 0.809, *p* = 0.003), FC (r = 0.664, *p* = 0.026), and total cholesterol (r = 0.756, *p* = 0.006) was significant. There were only four women, and the statistical test is not meaningful.

### 3.10. Urinary PCSK9 of Patients and Controls

**Figure 4:**
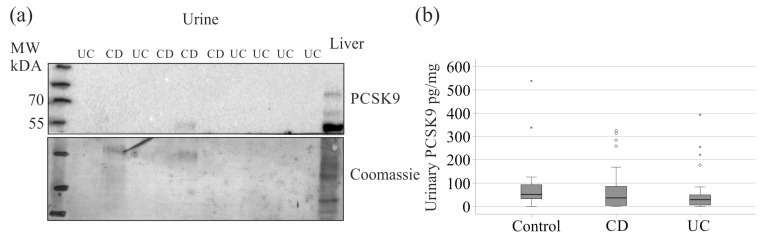
Urinary PCSK9. (**a**) The immunoblot used to detect PCSK9 in the urine of 10 patients with IBD. Liver tissue was used as positive control. The Coomassie-stained membrane is shown as a loading control. Molecular weight, MW; (**b**) urinary PCSK9 levels normalized to urinary creatinine of controls, patients with Crohn’s disease (CD), and patients with ulcerative colitis (UC). Statistical test: Kruskal–Wallis test with Bonferroni correction. Small circles and asterisks in the figures are outliers.

Urinary metabolites have long served as biomarkers [53,54], and this study assessed PCSK9 in urine of patients and controls. Immunoblot analysis of urinary samples from 10 IBD patients detected PCSK9 in one sample (Figure 4a). Therefore, an ELISA was used to determine urinary PCSK9 levels. Among 74 patients with measured plasma PCSK9, urinary PCSK9 levels were 33 pg/mL (0–377), significantly lower than serum levels, which were 295 (90–2090) ng/mL or approximately 10,000 times higher.

For analysis, urinary PCSK9 was normalized to urinary creatinine. Levels of urinary PCSK9 in CD (49 patients), UC (25 patients), and 17 controls showed no significant differences (*p* = 0.110) (Figure 4b). Urinary PCSK9 did not correlate with serum PCSK9, age, CRP, calprotectin, AST, ALT, GGT, bilirubin, AP, serum creatinine, FC, or total cholesterol. Positive correlations were observed between urinary PCSK9 and serum CE species 14:0 (r = 0.280, *p* = 0.046), 18:3 (r = 0.312, *p* = 0.026), 20:3 (r = 0.331, *p* = 0.018), 22:4 (r = 0.305, *p* = 0.029), and 22:5 (r = 0.440, *p* = 0.001).

In females, urinary PCSK9 correlated with CE 22:4 (r = −0.471, *p* = 0.029) and with CE 22:5 (r = 0386, *p* = 0.047) in males.

## 4. Discussion

This study is the first to look at PCSK9 alongside FC and CE species levels in male and female patients with IBD. Our results show that FC and CE species levels decrease with increasing disease activity in males, while PCSK9 levels remain in the normal range of both sexes. The homogeneous decrease in the different CE species and FC suggests that pathways affecting overall cholesterol homeostasis, rather than species-specific pathways, are impaired in male patients with active IBD.

In a previous study, elevated serum PCSK9 levels were observed in UC patients with high fecal calprotectin levels compared to UC patients with almost normal fecal calprotectin levels [24]. Serum PCSK9 was also found increased in UC patients compared to healthy controls [55]. In the present cohort, PCSK9 levels were found to be within the normal range for patients with IBD. No associations were observed between PCSK9 levels and serum CRP or fecal calprotectin. Furthermore, patients with CD and UC exhibited comparable levels to healthy controls, indicating that these two disease entities are not distinguishable. The patients with UC enrolled by Marinelli and coworkers had nearly 3-fold higher fecal calprotectin levels in comparison to the current cohort [24]. The median CRP level of the patients included by Deng and coworkers was 40.7 mg/L and was 2 mg/L in our cohort [55]. This indicates that the increased inflammation of the patients enrolled in these recent studies may have contributed to higher PCSK9 levels [24,55]. However, it is difficult to judge whether our patients indeed had less severe disease because calprotectin assays are not yet standardized [56].

Hypercholesterolemia is associated with high serum PCSK9 levels [23]. In the cohort of Marinelli et al., almost 60% of patients had total cholesterol levels > 200 mg/dL, which is consistent with higher serum PCSK9 levels [24]. In our patient cohort, 9% of the patients had hypercholesterolemia, and the total cholesterol levels of controls and patients were comparable. Deng et al. compared PCSK9 in controls and patients with IBD, but did not analyze serum cholesterol levels [55]. High serum cholesterol levels are associated with increased PCSK9 and need to be taken into account in studies evaluating PCSK9 as a biomarker [24,55].

However, the decrease in PCSK9 during effective therapy in UC patients [55], which may be associated with normalization of cholesterol levels [50], cannot be explained by the inclusion of patients with hypercholesterolemia [55].

PCSK9 is highly abundant in the liver, and patients with liver disease may have elevated serum levels [23]. In a cohort comprising nearly 700 individuals, positive correlations were observed between plasma PCSK9 levels and ALT, AST, alkaline phosphatase, and GGT [57]. The two previous studies that measured PCSK9 in patients with UC did not report liver enzyme levels in patients with UC [24,55] to exclude chronic liver injury as a confounding factor. In our cohort, modest correlations were mostly observed between PCSK9 levels and markers of liver disease in patients with IBD of both sexes. The patients in our study had relatively normal liver function. It should also be noted that patients with primary scleosing cholangitis and underlying IBD were excluded from our cohort. Interestingly, the correlations between liver enzymes and CE species levels were positive in men and negative in women. Due to the high *p*-values, these observations need to be confirmed.

A study in rats indicated the inhibition of colonic PCSK9 as a potential strategy for the management of IBD. This study described higher PCSK9 protein levels in the colon of rats with IBD compared to controls [26]. Serum PCSK9 levels were not analyzed in this study, and PCSK9 expressed in the colon seems not to contribute to circulating levels [23,26]. At present, data on serum levels of PCSK9 in IBD are inconsistent and analyses of well-described, large cohorts that can correct for confounding factors such as hypercholesterolemia and severe liver disease are needed.

The most thoroughly researched function of PCSK9 is its role in regulating serum LDL-cholesterol levels. A paucity of studies has analyzed the correlations between PCSK9 and CE species levels. It has been demonstrated that patients with PCSK9 loss-of-function exhibit diminished levels of CE 16:0, 18:1, and 18:3 [58]. In patients with moderate coronavirus disease 2019, serum PCSK9 exhibited a positive correlation with CE 18:3 and CE 20:5 [59]. In patients with IBD, positive correlations were observed between PCSK9 and CE 14:0, 14:1, 15:0, 15:1, 16:1, 18:1, 18:3, and 20:3. This shows that PCSK9 mostly correlated with ACAT-derived CE species, which are abundant in LDL [60].

The current analysis also showed that correlations of PCSK9 with several of these CE species were significant in males but not in females. PCSK9 accounts for less than 8% of plasma LDL [22], and, in our analysis, total cholesterol levels were measured. Cholesterol is also transported in high-density lipoprotein, whose levels are higher in females [21,61]. However, the factors that contribute to different results in male and female patients still need to be investigated.

Previous analysis revealed that the blood cholesterol levels of patients with active IBD are reduced in comparison to healthy controls, whereas cholesterol levels of patients with quiescent IBD are quite normal [62]. This study did not analyze cholesterol levels and disease severity by sex [62]. Accordingly, in our whole cohort, total cholesterol, CE, and FC levels were similar between patients with IBD and controls. CE 22:5 levels in the serum of our CD and UC patients were lower than in controls and further study has to show whether CE 22:5, a very low abundant lipid species produced by LCAT [10], is of special interest in IBD. Notably, a decline of CE 22:5 was noticed in both sexes.

Sex-specific analyses showed that the decrease in CE species in active disease was male-specific. Most of the CE species showed a negative correlation with fecal calprotectin and serum CRP levels in male patients. FC levels were also reduced in male patients with severe IBD. The parallel decrease in FC and almost all CE species indicates disruption of key pathways for cholesterol homeostasis in males. Notably, levels of saturated, monounsaturated, and polyunsaturated CE species decreased with increased inflammation, indicating that this association is independent of fatty acid saturation. CE species produced by both LCAT (e.g., CE 20:4, 22:5, 22:6) and ACAT (e.g., CE 14:0, 16:0, 18:1, 18:3) were reduced in severe IBD in male patients [10,11,63], and further research is needed to determine if the activities of both of these enzymes decline with disease severity in male patients with IBD. There was no shift from unsaturated to saturated CE species, and all CE species were similarly reduced in male patients with active IBD. This rules out an accumulation of saturated CEs relative to unsaturated CEs contributing to the higher risk of atherosclerosis in patients with IBD [16].

We are aware of one study that compared cholesterol levels between male and female patients with IBD and found no disease-related differences [20]. However, this study did not analyze the association of lipids with disease severity [20]. The epidemiology, clinical course, and outcome of IBD differ between male and female patients [19]. Blood levels of LDL cholesterol are lower in premenopausal women than in men [18,21] but have not been analyzed in patients with active disease in either sex. In the present analysis, only total cholesterol was measured, which did not differ between the sexes in the entire cohort in accordance with previous findings [20]. In our cohort, male and female patients had similar CRP and fecal calprotectin levels, suggesting that the disease severity was comparable. There is currently no explanation for the sex-specific decrease in serum cholesterol levels in male patients with IBD. Of note, the male and female patient cohorts included in our analyses had almost identical numbers of patients.

The GSRS is a tool that assesses the severity of various symptoms associated with gastrointestinal disorders, including abdominal discomfort and diarrhea [64]. No significant decline in serum CE species levels was observed in patients with higher GSRS scores. This suggests that CE species levels are mostly associated with disease severity, explaining the negative correlations of CE species with GSRS scores in males, but not with disease symptoms.

PCSK9 and CE species levels remained unaltered in patients with CD and UC according to the disease localization. Cholesterol is predominantly absorbed in the upper small intestine [65], which was not affected in our patients. Furthermore, it remains unclear whether cholesterol uptake, cholesterol usage, or excretion are mostly disturbed by inflammation [66] and why there is a sex-specific effect in patients with IBD.

Bile acid synthesis from cholesterol is important for elimination [67]. The fecal levels of secondary bile acids positively correlated with FC, CE, and total cholesterol levels in UC but not CD patients. These correlations remained significant in the male patients with UC. The female subcohort was too small for statistical tests and whether secondary bile acid levels also associate with cholesterol remains to be studied. Levels of secondary bile acids declined with higher fecal calprotectin levels in patients with UC [46]. The decline of secondary bile acids is related to dysbiosis, which may contribute to lower serum cholesterol in UC [68]. However, correlations do not imply functional relationships, and no such associations were observed in patients with CD.

Urine metabolites are routinely used to assess renal function [69], and urine is also being used to discover new non-invasive biomarkers for IBD diagnosis [53]. The current analysis showed that urinary levels of PCSK9 are very low. Urinary PCSK9 levels were not associated with IBD severity. Urinary PCSK9 levels were very weakly positively correlated with serum CE species levels but not with serum PCSK9 levels, an observation that requires further confirmation. PCSK9 in the urine is increased in patients with proteinuria, and PCSK9 inhibition reduces albumin in the urine of nephrotic mice [70]. Our patients had fairly normal creatinine levels, indicating good kidney function. Normal renal function and normal serum levels of PCSK9 in patients with IBD are consistent with normal urinary levels of PCSK9.

It should be noted that this study is not without limitations. The patients were recruited from a city in the south of Germany and the surrounding areas, and, therefore, the results may not be valid for other populations. Serum samples were collected during the day without fasting. Recent trends favor non-fasting lipid profiles over fasting profiles, as studies show minimal differences in lipids, lipoproteins, and apolipoproteins between the two states—except for triglycerides, which are generally higher when non-fasting [71]. Dietary habits of patients and controls were not recorded, as individual and population differences make absolute fatty acid intake challenging to assess [72]. Due to the small number of patients, associations between site of disease and sex were not calculated.

## 5. Conclusions

In conclusion, our study shows that PCSK9 does not play a role in IBD-related inflammation, which is associated with hypocholesterolemia in male but not in female patients. The clinical course of IBD differs between male and female patients [19], and the association of almost all CE types with IBD severity in males represents another sex difference in IBD. However, confirmatory analyses in larger cohorts are needed.

## Figures and Tables

**Table 1:** The data for male and female patients are presented as median, minimum, and maximum values. Serum cholesterol levels were available for 53 patients, and these subcohorts are described separately (alanine aminotransferase (ALT), alkaline phosphatase (AP), aspartate aminotransferase (AST), gamma glutamyl transferase (GGT)). Statistical tests: Mann–Whitney U test. * *p* < 0.05, ** *p* < 0.01, *** *p* < 0.001.

Characteristics	Male IBD Patients	Female IBD Patients	Male IBD Patients for CE Analysis	Female IBD Patients For CE Analysis
Number	38	42	29	24
Age (years)	48.9 (19.1–66.9)	38.3 (19.1–69.9)	45.2 (20.0–66.9)	38.3 (19.1–69.9)
BMI (kg/m^2^)	24.9 (15.5–40.4)	23.4 (17.5–44.3)	24.7 (15.5–40.4)	22.8 (17.5–44.3)
C-reactive protein (mg/L)	1 (0–144)	2 (0–44)	2 (0–144)	2 (0–44)
Fecal calprotectin (µg/g)	78 (0–3889)	44 (0–1097)	78 (0–1616)	50 (0–1097)
AST (U/L)	25 (12–41)	23 (10–34)	26 (12–35)	23 (10–34)
ALT (U/L)	20 (9–63)	18 (7–45)	20 (9–63)	18 (7–45)
GGT (U/L)	33 (11–100) *	24 (10–67)	31 (11–100) **	20 (10–37)
AP (U/L)	69 (40–142)	62 (43–127)	70 (46–142)	60 (43–117)
Bilirubin (mg/dL)	0.5 (0.2–1.7)	0.4 (0.1–1.9)	0.4 (0.2–1.7)	0.4 (0.1–1.9)
Creatinine (mg/dL)	0.89 (0.69–1.18) ***	0.73 (0.51–1.00)	0.89 (0.72–1.14) ***	0.74 (0.51–0.89)

**Table 2:** Spearman correlation coefficients for the correlation of PCSK9 with total cholesterol levels, free cholesterol levels, and cholesteryl ester (CE) species levels as well as correlations with C-reactive protein (CRP) and fecal calprotectin in male and female patients with IBD. * *p* < 0.05, ** *p* < 0.01.

PCSK9 and Cholesterol Metabolites	PCSK9	CRP	Calprotectin	PCSK9	CRP	Calprotectin
	Male Patients			Female Patients		
PCSK9		−0.145	−0.049		0.186	0.197
CE 14:0	0.425 *	−0.457 *	−0.710 **	0.180	−0.414	−0.245
CE 14:1	0.486 **	−0.258	−0.536 **	0.268	−0.295	−0.115
CE 15:0	0.335	−0.402 *	−0.615 **	0.123	−0.355	−0.177
CE 15:1	0.486 **	−0.336	−0.595 **	0.028	−0.355	−0.045
CE 16:0	0.229	−0.487 **	−0.575 **	0.066	−0.095	−0.357
CE 16:1	0.382 *	−0.239	−0.428 *	0.209	0.124	−0.060
CE 18:1	0.343	−0.546 **	−0.518 **	0.295	−0.175	−0.320
CE 18:2	0.128	−0.533 **	−0.442 *	0.003	−0.265	−0.439 *
CE 18:3	0.380 *	−0.635 **	−0.666 **	0.265	−0.276	−0.417 *
CE 20:3	0.291	−0.486 **	−0.591 **	0.261	−0.229	−0.314
CE 20:4	0.229	−0.565 **	−0.388 *	−0.011	0.004	−0.339
CE 20:5	0.300	−0.543 **	−0.569 **	0.059	−0.253	−0.353
CE 22:4	0.278	−0.598 **	−0.331	−0.299	−0.143	−0.126
CE 22:5	0.364	−0.479 **	−0.476 **	−0.226	−0.286	−0.274
CE 22:6	0.153	−0.483 **	−0.324	−0.133	−0.293	−0.254
Free Cholesterol	0.384 *	−0.562 **	−0.459 **	0.076	0.153	−0.448 *
Total Cholesterol	0.362	−0.623 **	−0.505 **	0.029	−0.195	−0.418 *

**Table 3:** Spearman correlation coefficients for the correlation of total cholesteryl ester (CE) levels, free cholesterol, and total cholesterol levels with fecal bile acid levels in the whole cohort. ** *p* < 0.01, *** *p* < 0.001.

Cholesterol	CD			UC		
	Primary Bile Acids	Secondary Bile Acids	Total Bile Acids	Primary Bile Acids	Secondary Bile Acids	Total Bile Acids
Total CE	−0.191	0.272	−0.062	0.121	0.836 ***	0.807 ***
Free Cholesterol	−0.289	0.316	−0.074	0.125	0.725 **	0.721 **
Total Cholesterol	−0.191	0.272	−0.062	0.075	0.811 ***	0.775 ***

## Data Availability

The raw data supporting the conclusions of this article will be made available by the authors on request.

## Supplementary Materials

The following supporting information can be downloaded at: https://www.mdpi.com/article/10.3390/pathophysiology32020013/s1. Table S1. Spearman correlation coefficients for the correlation of PCSK9 with total cholesterol levels, free cholesterol levels and cholesteryl ester (CE) species levels as well as correlations with C-reactive protein, and fecal calprotectin in the whole cohort. Table S2. Spearman correlation coefficients for the correlation of the Bristol stool chart and the Gastrointestinal Symptom Rating Scale (GSRS) with PCSK9, total cholesterol levels, free cholesterol levels and cholesteryl ester (CE) species levels. Table S3. Spearman correlation coefficients for the correlation of PCSK9, free cholesterol, cholesteryl ester (CE) species and total cholesterol levels with alanine aminotransferase (ALT), alkaline phosphatase (AP), aspartate aminotransferase (AST), gamma glutamyl transferase (GGT) and bilirubin in the whole cohort.
