# Supplementary material for: Cholesteryl Ester Species but Not Serum Proprotein Convertase Subtilisin/Kexin Type 9 Levels Decline in Male Patients with Active Inflammatory Bowel Disease
Source: Pathophysiology. 2025 Mar 25;32(2):13. doi: 10.3390/pathophysiology32020013 (PMC12015773; doi:10.3390/pathophysiology32020013)
Supplement: Supplementary file 1 [file pathophysiology-32-00013-s001.zip › pathophysiology-3379362-supplementary.pdf]

Table S1. Spearman correlation coefficients for the correlation of PCSK9 with total cholesterol levels, free cholesterol levels and cholesteryl ester (CE) species levels as well as correlations with C-reactive protein, and fecal calprotectin in the whole cohort. * $P < 0.05$, ** $P < 0.01$.

PCSK9 and cholesterol metabolites	PCSK9	C-reactive Protein	Calprotectin
PCSK9		-0.096	0.106
CE 14:0	0.366**	-0.374**	-0.550**
CE 14:1	0.426**	-0.251	-0.423**
CE 15:0	0.291*	-0.338*	-0.439**
CE 15:1	0.308*	-0.312*	-0.408**
CE 16:0	0.183	-0.356*	-0.489**
CE 16:1	0.362**	-0.120	-0.360**
CE 18:1	0.339*	-0.421**	-0.437**
CE 18:2	0.143	-0.415**	-0.457**
CE 18:3	0.356**	-0.472**	-0.575**
CE 20:3	0.302*	-.0378**	-0.499**
CE 20:4	0.113	-0.360*	-0.347*
CE 20:5	0.225	-0.429**	-0.477**
CE 22:4	-0.037	-0.428**	-0.222
CE 22:5	0.138	-0.431**	-0.370**
CE 22:6	0.042	-0.424**	-0.300*
Free Cholesterol	0.276*	-0.384**	-0.427**
Total Cholesterol	0.294**	-0.368**	-0.278*

Table S2. Spearman correlation coefficients for the correlation of the Bristol stool chart and the Gastrointestinal Symptom Rating Scale (GSRS) with PCSK9, total cholesterol levels, free cholesterol levels and cholesteryl ester (CE) species levels. * $P < 0.05$, ** $P < 0.01$.

PCSK9 and cholesterol metabolites	Bristol Stool Chart Male Patients	Bristol Stool Chart Female Patients	GSRS Male Patients	GSRS Female Patients
PCSK9	0.058	0.081	-0.125	0.161
CE 14:0	-0.289	-0.146	-0.473*	0.005
CE 14:1	-0.163	0.005	-0.499**	-0.026
CE 15:0	-0.345	-0.059	-.0419*	-0.028
CE 15:1	-0.234	-0.109	-0.442*	-0.122
CE 16:0	-0.441*	-0.051	-0.468*	0.063
CE 16:1	-0.206	0.262	-0.344	-0.121
CE 18:1	-0.477**	0.067	-0.453*	0.050
CE 18:2	-0.694**	-0.150	-0.507**	0.234
CE 18:3	-0.423*	-0.216	-0.402*	0.006
CE 20:3	-0.386*	-0.065	-0.451*	0.091
CE 20:4	-0.483**	-0.152	-0.356	-0.098
CE 20:5	-0.521**	-0.321	-0.433*	-0.045
CE 22:4	-0.504**	-0.291	-0.386*	-0.125
CE 22:5	-0.299	0.076	-0.461*	-0.085
CE 22:6	-0.504**	-0.332	-0.289	0.080
Free Cholesterol	-0.514**	- 0.010	-0.499**	-0.005
Total Cholesterol	-0.532**	-0.087	-0.544**	0.124

Table S3. Spearman correlation coefficients for the correlation of PCSK9, free cholesterol, cholesteryl ester (CE) species and total cholesterol levels with alanine aminotransferase (ALT), alkaline phosphatase (AP), aspartate aminotransferase (AST), gamma glutamyl transferase (GGT) and bilirubin in the whole cohort.

PCSK9 and cholesterol metabolites	ALT	AST	GGT	AP	Bilirubin
PCSK9	-0.137	-0.189	-0.046	0.035	0.150
CE 14:0	0.167	0.022	0.031	-0.036	0.141
CE 14:1	0.179	0.043	0.102	-0.001	0.065
CE 15:0	0.023	-0.107	-0.178	-0.140	-0.006
CE 15:1	0.109	0.003	-0.090	-0.214	-0.036
CE 16:0	0.234	0.116	0.112	0.141	-0.005
CE 16:1	0.150	0.034	0.150	0.152	-0.056
CE 18:1	0.179	0.030	0.128	0.098	-0.087
CE 18:2	0.252	-0.001	-0.150	0.005	0.060
CE 18:3	0.310	0.028	0.006	0.118	0.053
CE 20:3	0.265	0.178	0.213	0.188	0.093
CE 20:4	0.326	0.115	0.036	0.172	-0.142
CE 20:5	0.243	-0.033	-0.114	0.063	0.040
CE 22:4	0.225	-0.001	-0.109	0.054	-0.205
CE 22:5	0.242	0.045	0.089	0.230	-0.138
CE 22:6	0.235	-0.068	-0.226	0.023	-0.021
Free Cholesterol	0.197	0.035	0.079	0.193	-0.035
Total Cholesterol	0.238	0.141	0.118	0.038	0.119
